# Compression of the Ulnar Nerve Following Carpal Tunnel Release

**DOI:** 10.1177/22925503231190927

**Published:** 2023-08-01

**Authors:** Liam Robbins, Andrew Lovy, Joshua Gillis

**Affiliations:** 1 Memorial University School of Medicine, St. John's, Newfoundland, Canada; 2 Division of Orthopedic Surgery, Holy Cross Orthopedic Institute Fort Lauderdale, Fort Lauderdale, FL, USA; 3 Division of Plastic and Reconstructive Surgery, 7512 Memorial University, St. John's, Newfoundland, Canada

**Keywords:** carpal tunnel syndromes, Guyon syndromes, nerve compression syndromes, postoperative complications, syndromes du canal carpien, syndromes de Guyon, syndromes de compression nerveuse, complications postopératoires

## Abstract

Given the proximity and shared structures of Guyon's canal and the carpal tunnel, compression of the ulnar nerve is a rarely observed but possible complication of carpal tunnel release. In this case report, a patient underwent previous carpal tunnel release and immediately experienced ipsilateral hand weakness in keeping with an ulnar nerve compression syndrome. Clinical, electrodiagnostic, and magnetic resonance imaging findings after carpal tunnel release demonstrated a compression or injury to the deep motor branch of the ulnar nerve not previously present. Subsequent release of Guyon's canal identified a separate compartment of the deep motor branch of the ulnar nerve within the ulnar leaflet of the transverse carpal ligament. After the release of the motor branch from this compartment, the patient experienced recovery from their neuropathic symptoms. This case report outlines the relevant anatomy and clinical data surrounding an anomalous compartment of the deep motor branch of the ulnar nerve.

## Résumé

Compte tenu de la proximité et des structures partagées du canal de Guyon et du canal carpien, la compression du nerf ulnaire est une complication rarement observée mais possible de la libération du canal carpien. Dans ce rapport de cas, un patient a subi une libération du canal carpien, puis a éprouvé immédiatement une faiblesse de la main homolatérale, compatible avec un syndrome de compression du nerf ulnaire. Les constatations cliniques, électrodiagnostiques et d’imagerie de résonance magnétique après la libération du canal carpien ont montré une compression ou lésion de la branche motrice profonde du nerf ulnaire qui n’existait pas antérieurement. La libération subséquente du canal de Guyon a identifié un compartiment séparé de la branche motrice profonde du nerf ulnaire dans le feuillet ulnaire du ligament transverse du carpe. Après libération de la branche motrice de ce compartiment, le patient a guéri de ses symptômes de neuropathie. Cette étude de cas souligne l’anatomie et les données cliniques pertinentes entourant un compartiment anormal de la branche motrice profonde du nerf ulnaire.

A 70-year-old female developed a right-hand weakness immediately following a mini-open carpal tunnel release via a single incision in the distal palm. Inspection of the hand 3 months postoperatively demonstrated wasting and clawing of the interossei, and a positive Froment's sign. She had British Medical Research Council (BMRC) 0 of her dorsal interosseous. She had BMRC 4 abductor digiti minimi (ADM) function and normal power of the flexor carpi ulnaris and the flexor digitorum profundus to the ring and little finger.

Electrodiagnostic studies were conducted prior to and following release of the carpal tunnel. Prior to release, motor nerve conduction studies (NCS) yielded normal latencies, amplitudes, and velocities of the right radial and ulnar nerves. Motor responses were absent in the right median nerve. Electromyography (EMG) demonstrated fibrillations and severe chronic neurogenic changes to the abductor pollicis brevis (APB). Sensory NCS showed prolonged latency and decreased amplitude in the right ulnar nerve, in addition to an absence of the median nerve response. These findings were diagnostic of a right median neuropathy.

Four months after the release of the carpal tunnel, motor responses were present in the right median nerve. Normal motor responses were found in the right ulnar nerve when recording from the ADM, but a decreased amplitude was found when recording from the FDI. EMG showed active denervation and fibrillation potentials in the right adductor pollicis, APB, and FDI. No denervation was observed in the right ADM. Given these findings, in conjunction with the patient's clinical presentation, an injury or compression of the deep motor branch of the ulnar nerve was suspected.

**Figure 1.**
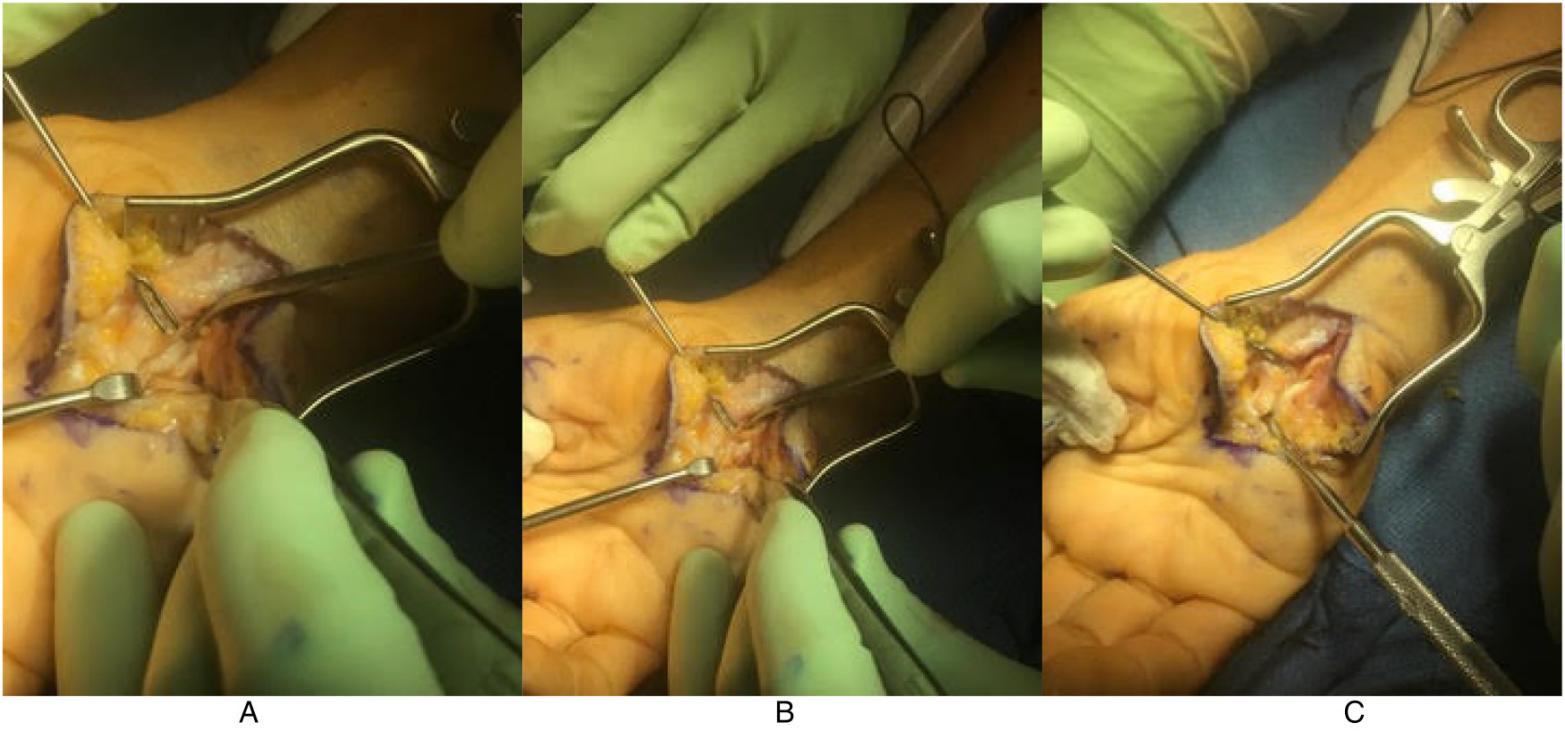
Isolation of the deep motor branch of the ulnar nerve prior to decompression. The separate compartment of the deep motor branch within the substance of the transverse carpal ligament can be seen. (A) The deep motor branch can be seen entering the compartment within the transverse carpal ligament. (B) the tenotomy scissors can be seen in the separate canal, alongside the deep motor branch, prior to complete release. (C) The compartment is demonstrated after mobilization of the deep motor branch.

**Figure 2.**
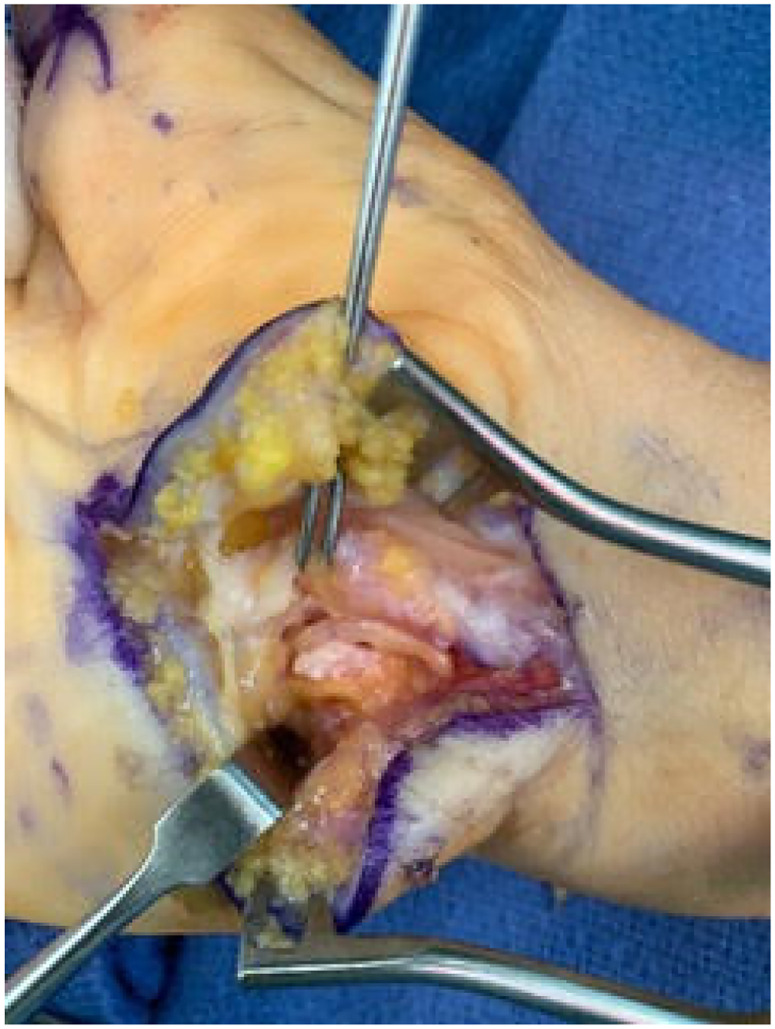
Deep motor branch of the ulnar nerve following decompression but lying within the separate compartment that arose within the transverse carpal ligament.

**Figure 3.**
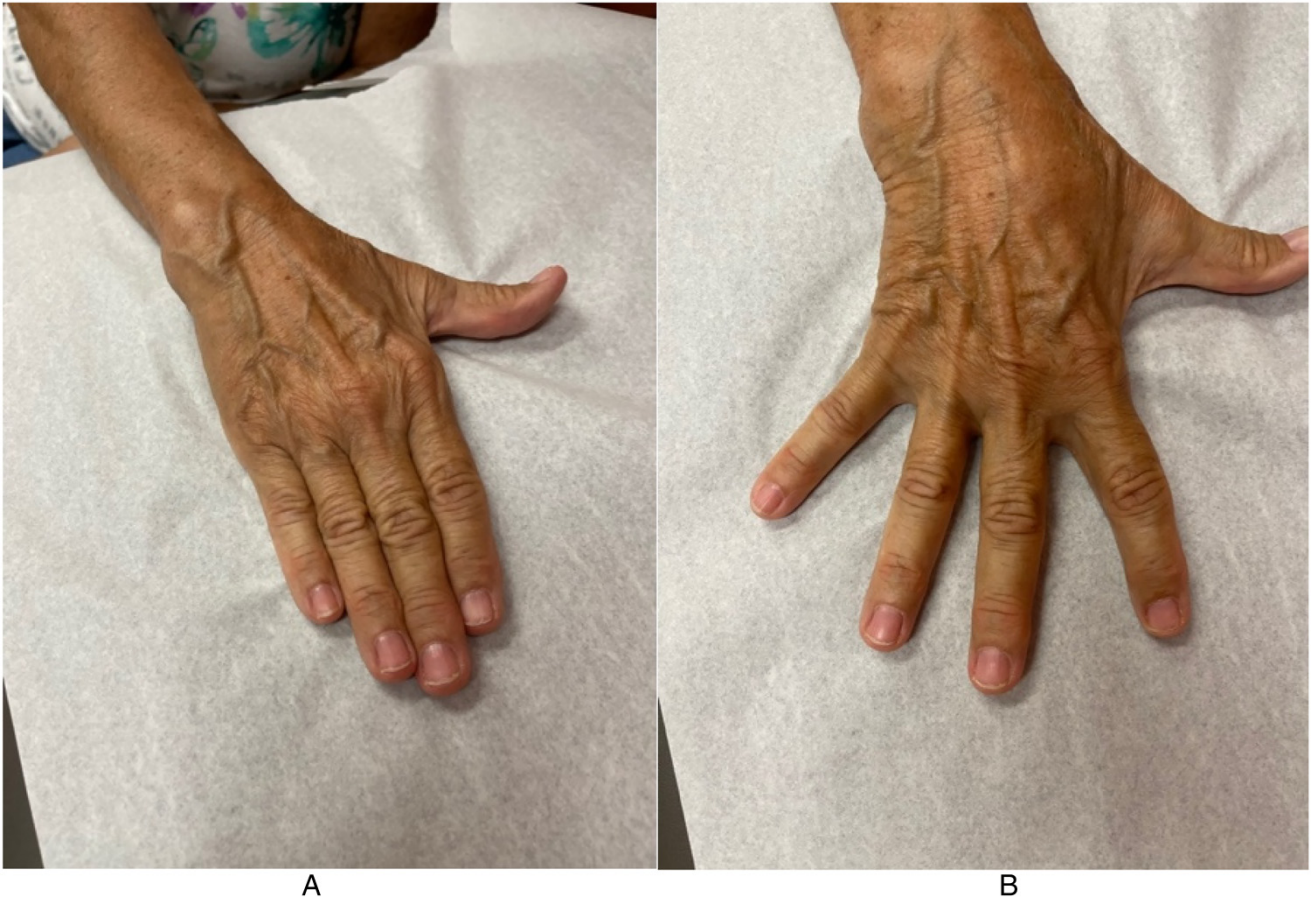
Postoperative hand function demonstrating full adduction (A) and abduction (B) of the digits.

A revision open carpal tunnel release and Guyon's canal release were then performed 6 months after the initial surgery. During the release of the deep motor branch of the ulnar nerve, a separate compartment of the deep motor branch was found within the substance of the transverse carpal ligament (Figure 1). After complete release, the deep motor branch was found to be intact throughout its course without injury (Figure 2). There was no response to intraoperative stimulation of the deep motor branch, in contrast to a positive response from the adjacent ADM branch. Twelve months after revision release, the patient demonstrated improved wasting, absent clawing of the interossei, and a negative Froment's sign. She had BMRC 5 in her first second and third dorsal interosseous and ADM (Figure 3).

Given the proximity to the carpal tunnel, this area is at risk of injury or compression during or after a carpal tunnel release.^1–3^ However, in our patient, there was a separate compartment of the deep motor branch within the transverse carpal ligament. Thus, when the transverse carpal ligament was released during the initial carpal tunnel release and allowed to retract, the deep motor branch of the ulnar nerve was compressed, resulting in weakness and denervation of the resultant intrinsic muscles. The ADM was spared as it branched prior to this compartment. Release of the deep motor branch led to full recovery of her ulnar intrinsic hand function.
